# Monocyte to High-Density Lipoprotein Ratio (MHR) as a predictor of mortality and Major Adverse Cardiovascular Events (MACE) among ST Elevation Myocardial Infarction (STEMI) patients undergoing primary percutaneous coronary intervention: a meta-analysis

**DOI:** 10.1186/s12944-020-01242-6

**Published:** 2020-03-26

**Authors:** Danielle Louis E. Villanueva, Marc Denver Tiongson, John Daniel Ramos, Elmer Jasper Llanes

**Affiliations:** Division of Cardiovascular Medicine, University of the Philippines, Philippine General Hospital, Taft Avenue, 1000 Manila, Philippines

**Keywords:** Monocyte to HDL ratio, MHR, STEMI, Primary PCI

## Abstract

**Background:**

Monocyte to High Density Lipoprotein Ratio (MHR) is a new marker that has been associated with major adverse cardiovascular outcomes among STEMI patients. We sought to strengthen the association between MHR and mortality and major adverse cardiovascular events (MACEs) among STEMI patients who underwent primary percutaneous coronary intervention.

**Methods:**

Studies were included if they satisfied the following criteria:1) Observational Studies; 2) Adult patients with ST-elevation Myocardial Infarction (STEMI) who underwent primary percutaneous intervention (PCI); and 3) Reported data on mortality and major adverse cardiovascular events. Using MEDLINE, Clinical Key, Science Direct, Scopus, and Cochrane Central Register of Controlled Trials databases, a search for eligible studies was conducted until September 2017. Our primary outcome of interest was all-cause cardiovascular (CV) mortality. We also investigated the association between MHR and major adverse cardiovascular events (MACEs).

**Results:**

We identified 3 studies involving 2793 STEMI patients, showing that in STEMI patients who underwent primary PCI, a high admission MHR is associated with a significantly higher in-hospital mortality [RR 4.71, (95% CI 2.36 to 9.39, *p* < 0.00001] and in-hospital MACE [RR 1.90, (95% CI 1.44 to 2.50), *p* < 0.00001]. This significant association was not observed in long term mortality or MACE.

**Conclusion:**

A high admission MHR among STEMI patients who underwent primary PCI is associated with a higher in-hospital mortality and MACE. This novel marker can be used as an inexpensive and readily available tool for risk stratification.

## Introduction

Atherosclerotic cardiovascular disease (ACD) is the most frequent underlying cause of coronary artery disease and it includes two major conditions: ischemic heart disease (IHD) and cerebrovascular disease. IHD and stroke are the world’s first and third causes of death, respectively, causing 247.9 deaths/100,000 persons in 2013, representing 84.5% of cardiovascular deaths and 28.2% of all-cause mortality worldwide [1]. Endothelial cells, leukocytes, and the intimal smooth muscle cells are the major components in the formation of an atherosclerotic plaque [2]. Inflammation plays an important role in the progression of atherosclerosis and cardiovascular diseases [3–7]. Atherosclerotic plaque rupture is the main culprit in the pathophysiology of acute ST-segment elevation myocardial infarction (STEMI) [8–10]. Monocytes are involved in the inflammatory response and contributes to the pathophysiology of all stages of atherosclerosis. Activated monocytes release circulating pro-inflammatory cytokines which can inflict damage to the to the elastic lamina and can predispose to atherosclerotic plaque rupture [11, 12]

In contrast, high density lipoprotein cholesterol (HDL-C) have anti-inflammatory, anti-oxidant, and anti-thrombotic effects [13, 14]. HDL-cholesterol protects endothelial cells from inflammation and oxidative stress by preventing monocyte recruitment into the artery wall by and by controlling monocyte activation and proliferation of monocyte progenitor cells [15–18].

It can be then hypothesized that there may be a relationship between a high monocyte count and a low HDL-C level in relation to the development and progression of atherosclerosis, and hence cardiovascular events. Association of high monocyte count and low HDL-C with inflammation and oxidative stress have led to testing a new marker called monocyte to high-density lipoprotein cholesterol ratio (MHR) in cardiovascular conditions. In fact, MHR may be superior to both the individual monocyte count and the HDL-C level in predicting short-term and long-term cardiovascular outcomes.

Recently, MHR has been reported to be a novel marker for major adverse outcomes in heart diseases [19–25]. It was first reported by Kanbay et al. [19] among CKD patients. They reported that patients with an increased MHR have higher fatal and non-fatal cardiovascular events, Hazard Ratio (HR) 2.24 and 4.91 respectively, compared to those with lower MHR. Canpolat and colleagues [20] reported that MHR was an independent predictor of atrial fibrillation recurrence after cryoballoon-based catheter ablation and was associated significantly with the presence of slow coronary flow.

Recent observational studies have focused on the association of MHR with mortality and major adverse cardiovascular events. In 2015, among patients with acute coronary syndrome who underwent PCI, admission MHR had a significantly positive correlation with neutrophil to lymphocyte ratio and CRP levels as an inflammation indicator. Patients with a higher MHR value had higher risk of in hospital mortality, long term MACE, stent thrombosis, and is associated with severity of coronary artery disease in the context of Gensini and Syntax scores [21, 22]. In the same study, a MHR cut-off of 14.43 had 80.2% sensitivity and 69.8% specificity for prediction of in-hospital MACE and a MHR value of 14.29 had a 81.5% sensitivity and 71.2% specificity for prediction of long term MACE.

Since most of the available literature is suggesting that MHR may be used as a new prognostic marker of cardiovascular events, we aim to synthesize the available evidence to determine the association of admission MHR with mortality and MACE among STEMI patients treated with primary PCI.

## Research question

Among adult STEMI patients treated with primary PCI, what is the association between monocyte-HDL ratio (MHR) and all-cause mortality and major adverse cardiovascular events (MACE)?

## Objectives

1. To determine the association between monocyte-HDL ratio (MHR) and all-cause mortality among STEMI patients treated with primary PCI.
2. To determine the association between monocyte-HDL ratio (MHR) and major adverse cardiovascular events (MACE) such as Ventricular Arrythmias (Ventricular Tachycardia or Ventricular Fibrillation), Reinfarction, Cardiopulmonary Resuscitation, and Target vessel revascularization among STEMI patients treated with primary PCI.

## Methodology

We conducted a meta-analysis following the proposed reporting guidelines of the Meta-analysis for Observational Studies in Epidemiology (MOOSE) group.

### Selection criteria

Studies were included in the meta-analysis if they satisfied the following inclusion criteria: 1) Observational Studies; 2) Adult patients with ST-elevation Myocardial Infarction (STEMI) who underwent primary percutaneous intervention (PCI); and 3) Reported data on mortality and major adverse cardiovascular events in association with the Monocyte to HDL ratio (MHR). Studies among patients with Stable CAD, Unstable Angina or Non-ST Elevation Myocardial Infarction, studies where primary PCI was not done, studies involving STEMI patients with previous PCI and studies with different outcomes of interest will be excluded.

### Literature search

The three authors of this review independently performed a systematic computer search in the Pubmed, MEDLINE, Clinical Trials, and Cochrane Central Register of Controlled Trials databases for eligible studies (May to September 2017) using a combination of the following search terms: “monocyte”, “High density lipoprotein”, “HDL”, “monocyte to HDL ratio”, “ST elevation myocardial infarction” “STEMI patients”, “primary percutaneous coronary intervention”, “primary PCI”, “major adverse cardiovascular events”, and “cardiovascular mortality”. No language, data, or publication restrictions will be imposed. Manual review of reference lists and journals were conducted to search for other eligible studies.

### Data collection and quality assessment

The identified studies were evaluated independently by the authors to determine their relevance for full text retrieval. The eligibility of the studies was then assessed by two independent reviewers (DLV and MDT) according to the specified inclusion criteria. Each study that fulfilled the inclusion criteria was assessed of methodological quality. Reasons for exclusion were documented. The Newcastle-Ottawa Scale (NOS) [26] was used to assess the quality of studies Using the NOS tool, a study may be awarded a maximum of nine stars to indicate high quality, and it makes use of three major domains, namely selection of the study groups, comparability of the two groups, and assessment of outcome. Disagreements were resolved by discussion with a third author. The author/s, year published, study design, population characteristics, sample size, outcomes (all cause or cardiovascular Mortality and MACE), and follow-up duration were extracted from each study. Disagreements were again resolved by discussion with the third author.

### Data analysis

Meta-analysis was conducted using the Mantel-Haenzel random-effects model to generate risk ratios, 95% confidence intervals (CIs), and forest plots. Heterogeneity was assessed through the chi square and I^2^ test, and pre-specified subgroup and sensitivity analysis were performed. For subgroup analysis, the included studies were categorized according to study design (retrospective versus prospective). We also did an analysis for the studies that presented long-term data for our outcomes of interest. Sensitivity analysis was done to evaluate the stability of pooled estimates and check for significant change in results after individually excluding each of the studies. A study would be considered an outlier if its exclusion from the analysis produced a 95% confidence interval that did not overlap with the 95% confidence interval of the original pooled estimate that included all studies. Funnel plot analysis was used to evaluate for publication bias. All statistical analyses were carried out using Review Manager (RevMan) version 5.3 (The Nordic Cochrane Centre, The Cochrane Collaboration, Copenhagen).

## Results

### Output of literature search

**Fig. 1:**
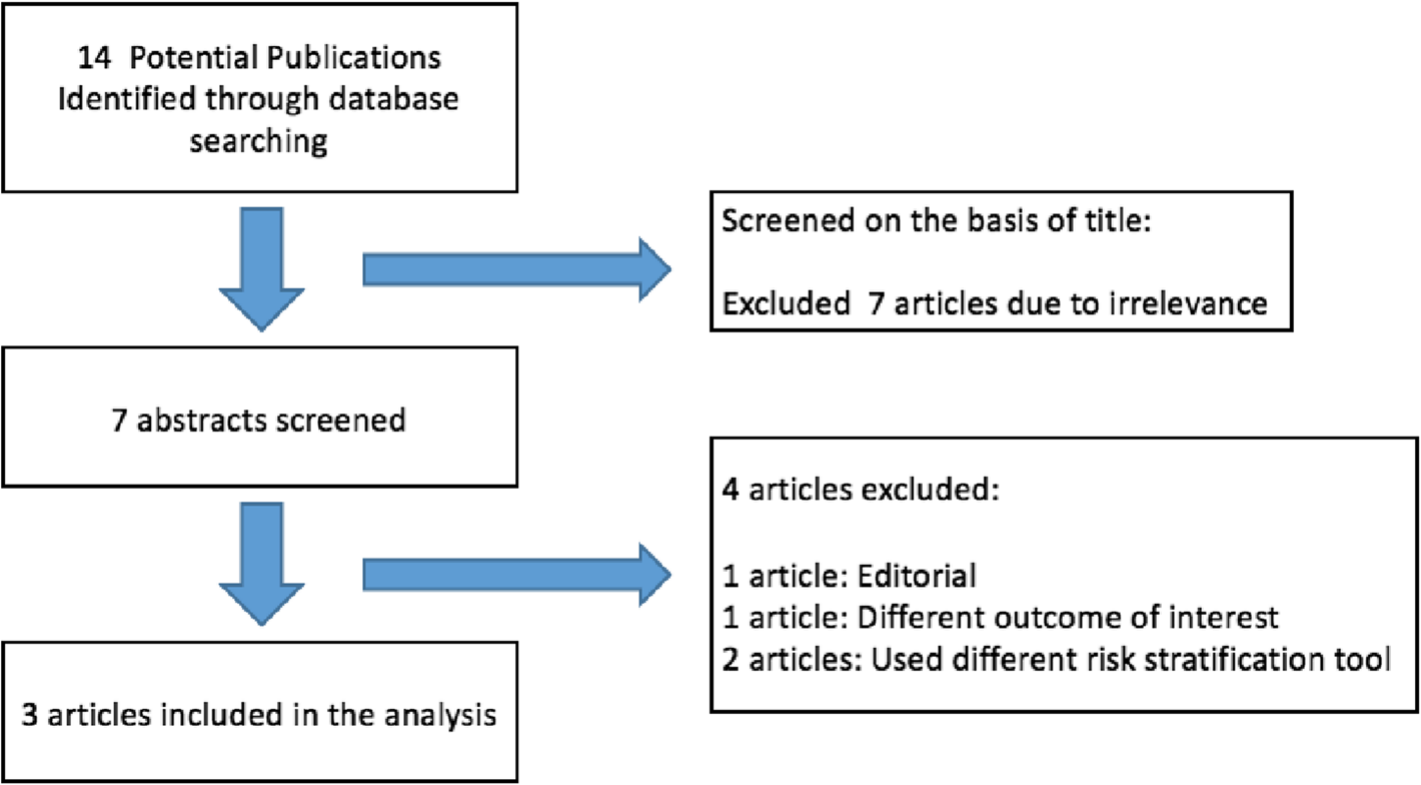
PRISMA Flow chart of Study Selection

Our systematic literature search yielded an initial 14 articles. Figure 1 shows the flow diagram of the search and identification process which included 3 articles comprising of 2793 STEMI patients eligible for this meta-analysis.

### Description of included studies

Table 1 summarizes the characteristics and details of the included studies. Two of out 3 included studies were prospective observational studies reported between 2015 and 2016. All studies were conducted in different tertiary training hospitals in Turkey. In the study by Karatas, the mean age was 56.4 ± 12.5 years with 67% males, in the study by Cicek, the mean age was 56.4 ± 12.4 years with 85% males, and in the study by Acikgoz, the mean age was 56.1 ± 11.8 years with 84% males. The participants were divided into different subgroups according to the value of their monocyte to HDL ratio (MHR). Only two studies (Acikgoz et.al and Cicek et.al) determined both short term and long term mortality and MACE as outcomes.

**Table 1:** Characteristics of Studies Included in the Meta-Analysis

Study Title, Study Design, Author, and Year of Publication	Population and Population Size	Study Design	Mean Monocyte to HDL ratio (MHR) groups	Mortality Outcome	MACEs Evaluated
Monocyte to high-density lipoprotein cholesterol ratio is predictive of in-hospital and five-year mortality in ST-segment elevation myocardial infarction ***Acikgoz et.al. 2016*** [24]	1598 patients with STEMI who underwent primary PCI from December 2009 to December 2013	Retrospective Cohort	*Tertile 1:* 533 patients with 8.63 ± 3.10 *Tertile 2:* 533 patients with 19.8 ± 3.8 *Tertile 3:* 532 patients with 30.1 ± 10.5	• *In Hospital Mortality* • *Long-term mortality* (after 60 month follow up)	• ***In-hospital MACE*** (Reinfarction, Stroke, Target vessel revascularization, Ventricular arrhythmia, Cardiopulmonary resuscitation and Death) • ***Long term MACE*** (Death, stroke, reinfarction and target vessel revascularization during 60-month follow-up)
Monocyte to high-density lipoprotein ratio as a new prognostic marker in patients with ST-segment elevation myocardial infarction undergoing primary percutaneous coronary intervention ***Karatas et.al. 2015*** [23]	513 STEMI patients who underwent primary PCI from January 2010 to January 2013	Retrospective Cohort	*Tertile 1:* 171 patients with 10.4 *Tertile 2:* 171 patients with 17.8 *Tertile 3:* 171 patients with 30.7	• *In-hospital mortality*	• ***In-hospital MACE*** (Ventricular arrhythmia, Reinfarction, Cardiopulmonary resuscitation, Target vessel revascularization, and Death during index hospitalization)
The relationship between admission monocyte HDL-C ratio with short-term and long-term mortality among STEMI patients treated with successful primary PCI ***Cicek et.al. 2016*** [25]	682 patients who underwent primary PCI from March 2013 to September 2015.	Prospective Design	*Quartile 1*: 172 patients with 8 ± 3 *Quartile 2*: 169 patients with 14 ± 1 *Quartile 3*: 161 patients with 19 ± 2 *Quartile 4*: 180 patients with 28 ± 7	• *In-hospital mortality* • *Long-term mortality*	• ***In-hospital MACE*** (Composite of death, nonfatal re-infarction, target vessel revascularization, or new- onset congestive heart failure during hospitalization) • ***Long Term MACE****at 30 month clinical follow-up*

*MACE* major adverse cardiovascular event, *STEMI* ST elevation myocardial infarction, *PCI* percutaneous coronary intervention

### Quality assessment of included studies

For quality assessment, two of the included studies (Acikgoz and Cicek) were given the highest rating of 9 stars on the Newcastle-Ottawa Scale, with the study by Karatas receiving on stars due to inadequate details on follow-up (Table 2).

**Table 2:** Quality Assessment of Included Studies Using Newcastle-Ottawa Scale

Author & Year of Publication	Selection Max 4	Comparability Max 4	Outcome	Total Rating
Acikgoz et.al. 2016 [24]	★★★★	★★	★★★	9★
Karatas et.al. 2015 [23]	★★★★	★★	★	7★
Cicek et.al. 2016 [25]	★★★★	★★	★★★	9★

### Results on the outcome of interest

#### MHR and mortality in STEMI patients

**Fig. 2:**
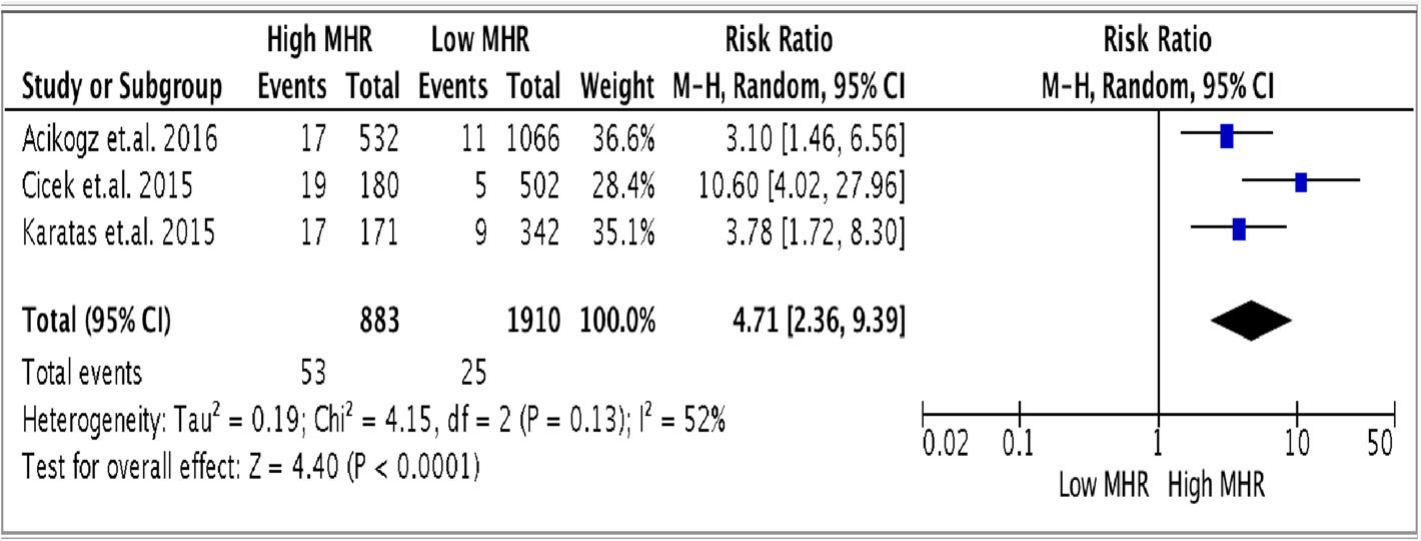
Forest plot showing pooled risk ratio of MHR and all cause mortality

The pooled risk ratio from the analysis of the 3 studies (Fig. 2) showed that a higher MHR value on admission of STEMI patients who underwent primary PCI is associated with a significantly higher all-cause in hospital mortality (6% for high MHR versus 1.3% for low MHR) [RR 4.71, (95% CI 2.36 to 9.39, *p* < 0.00001]. There was moderate degree of heterogeneity with an I^2^ value of 52%. On sensitivity analysis, the overall results remained robust and there were no studies removed as statistical outliers. To further investigate for heterogeneity, we proceeded with subgroup analysis.

**Fig. 3:**
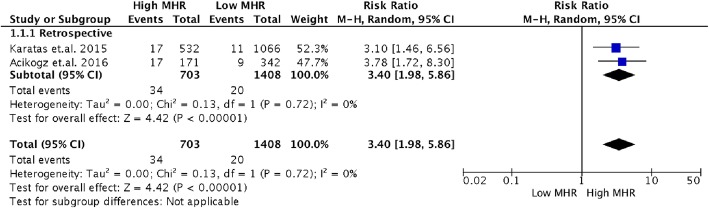
Subgroup analysis according to study design (Retrospective)

We classified the included studies according to the type of study design (Fig. 3). Two of the 3 included studies were retrospective studies. Pooled RR estimates have similar results to that of the original analysis [RR 3.4, (95% CI 1.98 to 5.86, *p* < 0.00001]. In the subgroup analysis, there was no significant heterogeneity (I^2^ = 0%).

#### MHR and in-hospital MACE in STEMI patients

**Fig. 4:**
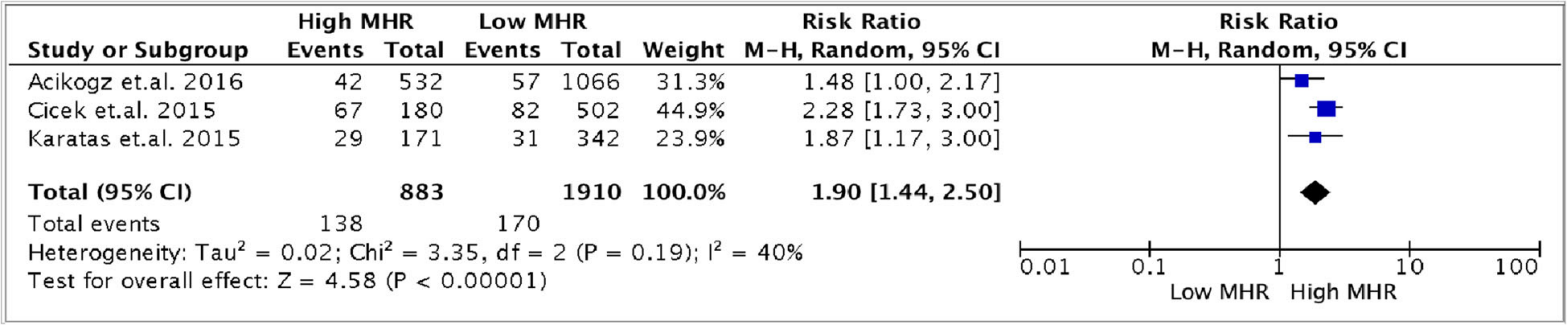
Forest plot showing pooled risk ratio of MHR and in-hospital MACE

In terms of in-hospital MACE, the pooled risk ratio (Fig. 4) also showed that a higher MHR value on admission of STEMI patients who underwent primary PCI is associated with a significantly higher occurrence of MACE (15.6% for high MHR versus 8.9% for low MHR) [RR 1.90, (95% CI 1.44 to 2.50), *p* < 0.00001]. There was no heterogeneity noted (*p* = 0.19, I^2^ = 40%).

**Fig. 5:**
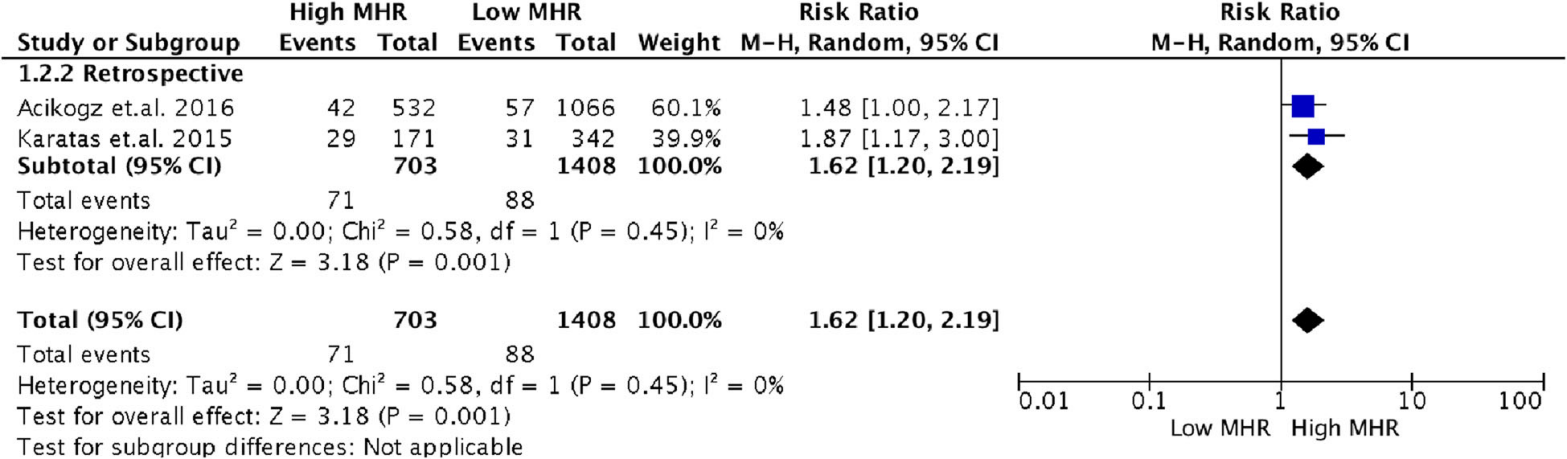
Subgroup analysis according to study design (Retrospective)

Subgroup analysis according to study design (Fig. 5) showed pooled RR estimates with similar results to that of the original analysis [RR 1.62, (95% CI 1.20 to 2.19, *p* < 0.00001]. The I^2^ value was lower (I^2^) compared to the original analysis.

#### MHR and long term all-cause mortality and MACE in STEMI patients

**Fig. 6:**
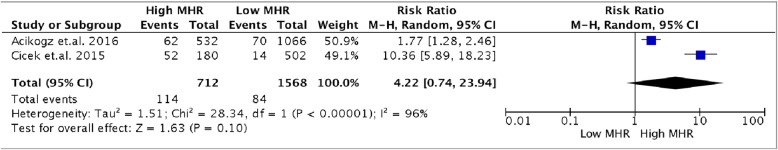
Forest plot showing pooled risk ratio of MHR and long term all cause mortality

**Fig. 7:**
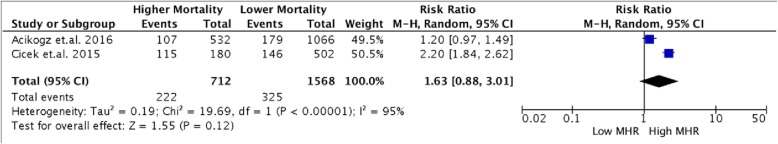
Forest plot showing pooled risk ratio of MHR and long term MACE

Only two studies determined long term outcomes. The pooled risk ratio (Figs. 6 and 7) showed that a higher MHR value on admission of STEMI patients who underwent primary PCI is associated with a non-significant higher occurrence of long term all-cause mortality (16% for high MHR versus 5.4% for low MHR) [RR 4.22, (95% CI 0.74 to 23.94), *p* < 0.00001] and long term MACE (31.1% for high MHR versus 20.7% for low MHR) [RR 1.63, (95% CI 0.88 to 3.01), *p* < 0.00001]. There was significant heterogeneity noted for both outcomes.

## Discussion

Previous studies have shown an association between high admission MHR with poorer cardiovascular outcomes among CKD patients [19], patients with atrial fibrillation [20], and ACS patients [22–25]. To the best of our knowledge, this is the first systematic review and meta-analysis demonstrating the impact of MHR with all cause mortality and major adverse cardiovascular events (MACE) among STEMI patients treated with primary PCI.

In this meta-analysis, it was showed that a high admission monocyte to HDL ratio (MHR) is associated with a higher risk of both in-hospital all cause mortality [RR 4.71, (95% CI 2.36 to 9.39)] and in-hospital MACEs [RR 1.90, (95% CI 1.44 to 2.50)]. Among STEMI patients treated with primary percutaneous coronary intervention, Karataş et al. [23], Acikgoz et.al [24]., and Cicek et.al [25] demonstrated that high admission MHR values ere independently associated with higher rates of in-hospital mortality and major adverse cardiovascular events such as ventricular arrhythmia (Ventricular Tachycardia or Fibrillation), re-infarction, cardiopulmonary resuscitation, and target vessel revascularization among patients with STEMI treated with primary PCI. In the study by Karatas et al. [23], analysis showed MHR greater than 17.1 as a cutoff value for mortality and MHR greater than 20.4 as a cutoff value for MACE.

In the analysis of long term outcomes, both showed a non-significant increased risk of all-cause mortality [RR 4.22, (95% CI 0.74 to 23.94)] and MACE [RR 1.63, (95% CI 0.88 to 3.01)]. Expectedly, there was significant heterogeneity for both analyses since the two studies had different follow-up duration (30 months versus 60 months). Another source of heterogeneity is the difference in study design of both studies (retrospective versus prospective). Furthermore, there was a wide confidence interval observed for both outcomes implying that the sample size included was too small.

The main pathophysiology behind acute STEMI is the rupture of an atherosclerotic plaque with subsequent activation of the coagulation cascade and thrombosis. Baseline pro-inflammatory and pro-oxidant status have been implicated as important predictors of adverse clinical outcomes in ACS [3–7]. Circulating monocytes account for the major source of pro-inflammatory and pro-oxidant factors and interact with endothelial cells and platelets leading to inflammation, thrombosis and endothelial dysfunction. In contrast, high density lipoprotein cholesterol (HDL-C) have anti-inflammatory, anti-oxidant, and anti-thrombotic effects [15, 16, 27]. HDL-cholesterol protects endothelial cells from inflammation and oxidative stress by preventing monocyte recruitment into the artery [27], by controlling monocyte activation and proliferation of monocyte progenitor cells [15, 16, 27] and by inhibiting oxidation of low density lipoprotein cholesterol [16].

The strength of this meta-analysis is that the selected patients in the included studies were homogenous with similar cardiovascular risk profiles relevant to STEMI. However, there was moderate heterogeneity which was improved by doing subgroup analysis. Study design was the major contributor of heterogeneity in terms of in-hospital outcome while the difference in follow-up duration played a major role for the long term outcomes. Another important possible source of heterogeneity is the classification of MHR categories. Two studies used MHR tertiles with different MHR cut-offs while the 3rd study used quartiles. There were no similar cut-offs for the MHR categories for the three studies although it was suggested by Karatas et.al that the MHR cut-off value for increased risk of mortality is 17.1 while the cut-off for MACE is 20.4.

Despite the significant correlation between admission MHR and in-hospital all-cause mortality and MACE, our findings do not suggest or imply a causal relationship. Our study has few limitations worth mentioning. First, despite observing significant association between MHR and in-hospital mortality and MACE, there was no similar definite MHR cut-offs for the three studies. We arbitrarily assigned the lowest tertile or quartile category as low MHR. Second, we were limited with the cardiovascular outcomes included as MACE as defined by the included studies. Some of the outcomes mentioned in the studies were clinically significant cardiovascular outcomes among STEMI patients, i.e. No-reflow, Development of Atrial Fibrillation, Use of intra-aortic balloon pump, but was not included in the final analysis. Lastly, two studies included patients who were on previous medications for dyslipidemia but the study by Karatas et.al excluded patients who were on chronic anti-hyperlipidemic agents.

With the evidence presented in this meta-analysis, we therefore recommend the conduct of prospective studies with larger sample size to further evaluate if there is indeed an association between admission MHR and cardiovascular outcomes. Future researches should clearly determine the specific MHR cut-off that would that would increase the risk of cardiovascular events. Another possible utility is monitoring of efficacy or response to therapy in relation to the MHR level. Since MHR is an inexpensive and readily available diagnostic test which is a component of routine laboratory examinations among our STEMI patients, we can look to its value in prognosticating cardiovascular outcomes.

## Conclusion

Our study has demonstrated that a higher admission MHR is associated with a higher risk for in-hospital mortality and MACE among STEMI patients who underwent primary PCI. However, despite the significant correlation, studies with larger sample size should be done to evaluate its clinical utility among STEMI patients undergoing primary PCI.

## Data Availability

The data used to support the findings of this study are included within the article. Additional data or information can be requested by contacting the corresponding author.

## Abbreviations

MHR: Monocyte to HDL Ratio
MACEs: Major Adverse Cardiovascular Events
STEMI: ST – Elevation Myocardial Infarction
ACS: Acute Coronary Syndrome
PCI: Percutaneous Coronary Intervention
CV: Cardiovascular
ACD: Atherosclerotic Cardiovascular Disease
IHD: Ischemic Heart Disease
HDL-C: High Density Lipoprotein Cholesterol
CKD: Chronic Kidney Disease
HR: Hazard Ratio
CI: Confidence Interval
CRP: C-Reactive Protein
MOOSE: Meta-analysis for Observational Studies in Epidemiology
NOS: New Castle Ottawa Scale
